# Effect of C and N Addition on Thermoelectric Properties of TiNiSn Half-Heusler Compounds

**DOI:** 10.3390/ma11020262

**Published:** 2018-02-08

**Authors:** Hwan Soo Dow, Woo Sik Kim, Weon Ho Shin

**Affiliations:** 1 Convergence R&D Division, Korea Institute of Ceramic Engineering and Technology, Jinju 52851, Korea; wskim@kicet.re.kr; 2 Energy Efficient Materials Center, Korea Institute of Ceramic Engineering and Technology, Jinju 52851, Korea

**Keywords:** thermoelectric material, half-heusler, thermal conductivity

## Abstract

We investigated the thermoelectric properties of the ternary half-Heusler compound, TiNiSn, when introducing C and N. The addition of C or N to TiNiSn leads to an enhanced power factor and a decreasing lattice thermal conductivity by point defect phonon scattering. The thermoelectric performances of TiNiSn alloys are significantly improved by adding 1 at. % TiN, TiC, and figure of merit (*ZT*) values of 0.43 and 0.34, respectively, can be obtained at 723 K. This increase in thermoelectric performance is very helpful in the commercialization of thermoelectric power generation in the mid-temperature range.

## 1. Introduction

The potential of thermoelectric (TE) materials and devices has been discussed in recent years due to their application possibility in power generation and electronic refrigeration fields [1,2]. The efficiencies of TE devices are highly dependent on the dimensionless figure of merit (*ZT*), which can be expressed as the following formula:

*ZT* = *S*^2^T/ρκ_T_ (1)

κ_T_ = κ_e_ + κ_ph_ (2)

where *S* is the Seebeck coefficient (or thermopower), ρ is the electrical resistivity, κ_T_ is the thermal conductivity, T is the absolute temperature, κ_e_ is electronic contribution of thermal conductivity, and κ_L_ is the lattice contribution of thermal conductivity [1]. Thus, good TE efficiencies and performances need a large *S* and small ρ and κ_T_ at each absolute temperature.

There are various types of TE materials for different operation temperatures. Bi_2_Te_3_-based compounds show good performance from room temperature to 200 °C [3,4], while SiGe-based compounds are suitable for high temperatures (over 800 °C) [5,6]. For the mid-temperature range (from 500 °C to 800 °C), lead chalcogenides, skutterudites, and half-Heusler compounds have been the most studied for TE devices. In spite of good TE efficiencies, lead chalcogenides include the toxic element Pb, and have poor mechanical properties; while skutterudites show weak thermal stabilities. On the other hand, half-Heusler compounds consist of environmental friendly elements, and show stable thermal and mechanical properties, which make them promising candidates for TE materials for the mid-temperature range [7,8,9,10,11,12].

In general, half-Heusler compounds consist of XYZ, where X could be a transition metal, a noble metal, or a rare-earth metal, Y could be a transition metal or noble metal, and Z is a main group of elements. The XYZ structure is a MgAgAs type (space group F-43m), where X, Y, and Z atoms consist of three interpenetrating FCC sub-lattices by occupying the Wyckoff positions of 4b(1/2, 1/2, 1/2), every other 4c(1/4, 1/4, 1/4) position is empty. Based on calculations, half-Heusler compounds have a valence electron count (VEC) of 18 per stable unit cell, and show a band-gap in the range of 0~1.1 eV, which is suitable for mid-temperature applications of TE devices [13,14,15,16,17,18,19,20].

Over the past few decades, half-Heusler intermetallic compounds (MNiSn: M = Zr, Hf, Ti) have been presented as highly promising TE materials due to their being small band-gap semiconductors (0.1~0.5 eV), their high *S* (200~300 μV/K) and their low ρ (0.1~8 mΩ·cm) [7,8,9,10]. Due to the simplicity of synthesis of TiNiSn-based compounds by addition or substitution of their constituents, there are many ways of optimizing their electronic and thermal properties. In spite of these advantages, the high κ_T_ (7~10 W/mK) issue of half-Heusler intermetallic compounds remains to be solved [7,8,9,10]. Additionally, half-Heusler intermetallic compounds consist of high melting point elements such as Zr, Hf, Ti, etc. Accordingly, high-temperature alloying methods are needed, such as arc-melting processes. To homogenize the melted alloy composition, high-temperature annealing (>800 °C) should be conducted after arc-melting for more than 72 h [21,22,23].

To overcome the thermal property issues, intensive research has been focused on the following points. First, phonon mass-fluctuation and the point defect scattering are adapted by heavy elements substitution, for example Ti(Zr, Hf)-Ni(Pd, Pt)-Sn [7,11,14,15,16]. Second, the fine grain size by powder metallurgy provides grain boundary scattering. However, the microstructural stability at high temperature is uncertain above 800 K. For the promotion of sufficient homogeneity and the ordering of the crystal structure, at least 1~2 weeks of annealing is required [17,18,19]. Third, nano-size particles or precipitates are applied in order to generate the phonon scattering center by large defects in the grains [7,20]. Lastly, interstitial atoms induce resonant scattering, which is the nonstationary nature of the impurity state, and loosely bond with their surrounds [1,24].

Additionally, it is also well known that the best figure of merit possessed by half-Heusler compounds, to date, is shared by two types of compounds. The first is a Zr-based half-Heusler: Zr_0.5_Hf_0.5_Ni_0.8_Pd_0.2_Sn_0.99_Sb_0.01_ shows *ZT* = 0.7 at 800 K using a solid-state reaction by SPS [25]; and (Zr_0.5_Hf_0.5_)_0.5_Ti_0.5_NiSn_0.998_Sb_0.002_ shows *ZT* = 1.5 at 700 K using hot-press at 1473 K [26]. The other is a Ti-based half-Heusler: Ti_0.95_Hf_0.05_NiSn_0.99_Sb_0.01_, *ZT* = 0.78 at 770 K using hot-press and annealing at 1023 K for 2 weeks [27]. In addition, recent research has reported state-of-the-art TE performances of *ZT* > 1 for half-Heusler alloys [8,28,29,30,31].

In this present work, a comprehensive investigation of the TiNiSn half-Heusler compounds was performed on the effect of C and N addition on TE properties in the temperature range from 323 K to 923 K. Most works on the TiNiSn compounds have been concentrated on the composition of TiNiSn by heavy element substitution. There are few studies on the investigation of the effect of the interstitial elements and annealing effects that could induce TE performance change of TiNiSn compounds. Thus, comprehensive studies of the TE properties of TiNiSn compounds with the interstitial atom, C and N, and the homogenization annealing are performed in this work. For TiNiSn compounds, the TE properties, including *S*, ρ, and κ_T_, were studied at elevated temperature.

## 2. Materials and Methods

Samples with the nominal composition of TiNiSn with TiC and TiN (TiNiSn + xTiC/yTiN (x, y = 0, 1, 10 at. %)) were prepared. Each element of Ti, Ni, Sn, TiC, and TiN granules (5N purity) was weighed and loaded into the arc-melting furnace chamber (DAIA vacuum engineering, Tokyo, Japan) with 10^−3^ Torr vacuum. The samples were flipped and remelted 2~3 times to ensure homogeneity. After arc melting, specimens were annealed at 900 °C for 24 h and 800 °C for 1 week, followed by furnace cooling. The obtained samples were cut into platelets and bars for characterization.

Structure identification was performed by x-ray diffraction (XRD, Rigaku D/max-rc (12 Kw), Rigaku, Tokyo, Japan) with Cu-Kα wavelength (1.5406 Å) in the range of 2θ between 20° and 80°. The densities of the samples were measured by the Archimedes method. The morphologies of the samples were analyzed using scanning electron microscopy (SEM, Hitachi S-4800, Hitachi, Tokyo, Japan). Thermoelectric properties of the electrical resistivity and the Seebeck coefficient were performed with ULVAC ZEM-3 (ULVAC, RIKO, Yokohama, Japan) in the temperature range from room temperature to 650 °C. Thermal conductivity at room temperature was performed by the static method. Thermal conductivity above room temperature was calculated from the values of thermal diffusivity measured by the laser flash method (NETZSCH, LFA-457, Selb, Germany). All measured TE transport data were obtained within the experimental error of *S* (~4%), ρ (~4%), and κ_T_ (~5%); thus, we assume total uncertainty of ZT as ~12%.

## 3. Results and Discussion

**Figure 1:**
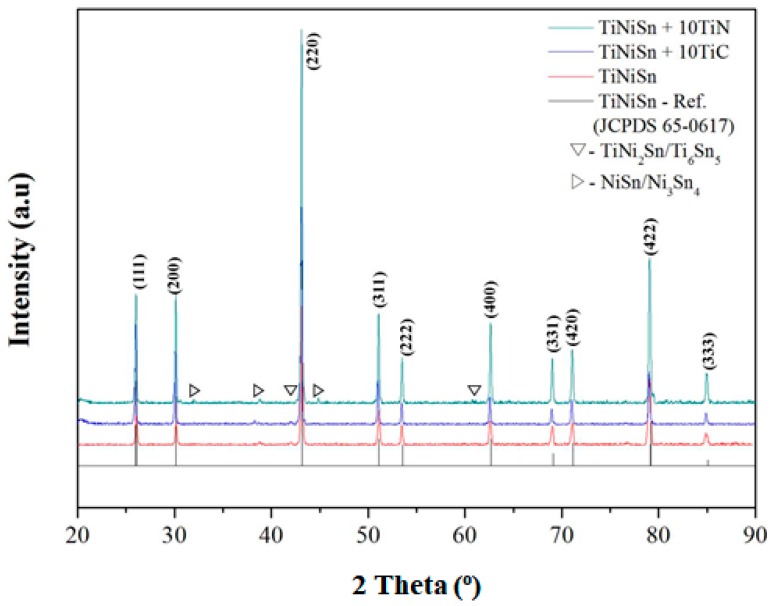
XRD (X-ray diffraction) patterns of arc-melted and annealed TiNiSn samples with the nominal compositions of TiNiSn, TiNiSn-10TiN, and TiNiSn-10TiC.

The XRD patterns of samples with the nominal composition x, y = 0 and x, y = 10 at. % are displayed in Figure 1. The XRD patterns revealed the existence of two secondary phases with small amounts of TiNi_2_Sn and NiSn impurities for the TiNiSn sample. Due to the similar crystal structure of those phases, they have the possibility to form TiNi_2_Sn, Ti_6_Sn_4_, and Ni_3_Sn_4_ phases in TiNiSn, which is similar to the other report [32]. This crystallization behavior is believed to arise from the stoichiometry of the half-Heusler in the Ti-Ni-Sn ternary system and the narrow phase composition of Ti-Ni-Sn ternary system [33].

**Figure 2:**
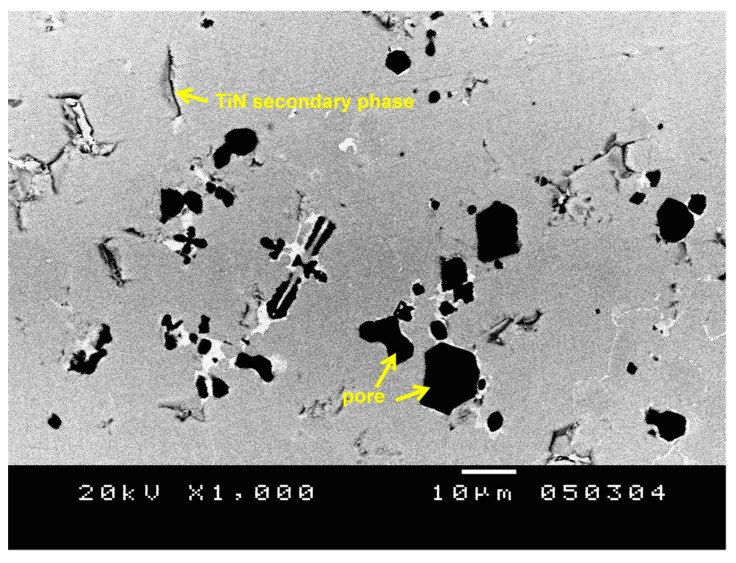
SEM BSE (scanning electron microscopy back-scattered electron) image of the surface of TiNiSn-10TiN alloy.

Figure 2 shows the SEM back-scattered electron (BSE) image of TiNiSn-10TiN alloy after melting and annealing. The pores (the black area in Figure 2) with the size of several μm could easily be found in the SEM image. This is because the samples in this work do not include the sintering process; however, the relative density reaches ~91%, which is high enough for use in thermoelectric applications. They also have some secondary phases of TiN due to the solubility limit of TiN in TiNiSn systems.

**Figure 3:**
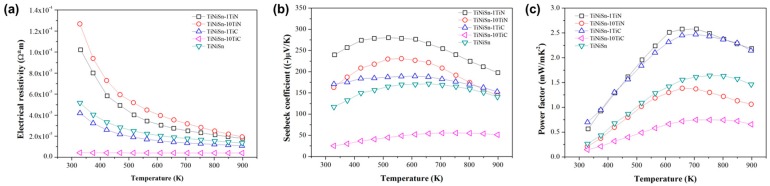
(**a**) The electrical resistivity, (**b**) the Seebeck coefficient, and (**c**) the power factor of TiNiSn, TiNiSn-1TiN, TiNiSn-10TiN, TiNiSn-1TiC, and TiNiSn-10TiC.

The ρ of TiNiSn and the corresponding TiC/TiN addition alloys was shown in Figure 3a. The temperature dependence of ρ for the samples of TiNiSn-xTiC and TiNiSn-yTiN is quite different. In the case of TiNiSn-xTiC compounds, the ρ showed a semiconducting (semi-metallic) behavior, which decreased with an increasing amount of additive of TiC, while the trend changed completely to a metallic property for TiNiSn-10TiC composition. On the other hand, the ρ of TiNiSn-yTiN was greatly increased with an increase in the amount of additive of TiN. The intrinsic semiconducting MNiSn alloy with 18 VEC (valence electron count) per unit cell has a typical electrical resistivity of 10^−1^~10^−3^ Ωcm at room temperature [34].

The temperature dependence of *S* of the TiNiSn compounds is shown in Figure 3b. The *S* values for all samples show negative, which means that the major carriers are electrons. The maximum *S* value is achieved with an addition of 1 at. % TiN to the TiNiSn alloy in all temperature ranges. The *S* for TiNiSn-1 at. % TiN alloy is maximized at the specific temperature, denoted hereafter as an onset temperature. The absolute values of the *S* are increased in the temperature range from the room temperature to the on-set temperature (region 1). After that, the *S* decreases above the on-set temperature (region 2). Region 1 could be comprehended as an extrinsic region for the degenerate semiconducting properties. Region 2 above the onset temperature could be understood as an intrinsic region. In region 2, the electrons are excited across the band-gap, and there is an increase in the concentrations of the holes as the temperature increases. As a result, the contribution of the holes to the total *S* is increased; the absolute value of the *S* is reduced. In n-type TiNiSn alloys, the onset temperature is absolutely defined and dependent on the concentrations of the carriers.

The temperature dependence of the power factors for TiNiSn alloys is shown in Figure 3c. The power factor can be calculated from the *S* and ρ using the equation of *S*^2^/ρ. The maximum power factor is achieved at the composition of TiNiSn-1 at. % TiN. The largest value in TiNiSn-1 at. % TiN is obtained 2.6 mW/mK^2^ at 700 K, mainly due to the large *S* value.

**Figure 4:**
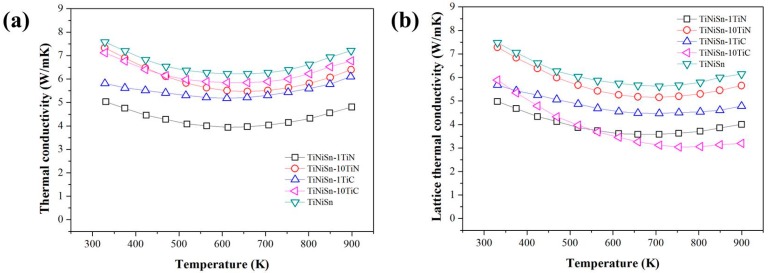
(**a**) The thermal conductivity and (**b**) lattice thermal conductivity of TiNiSn, TiNiSn-1TiN, TiNiSn-10TiN, TiNiSn-1TiC, and TiNiSn-10TiC.

The temperature dependence of the κ_T_ for the TiNiSn alloys is shown in Figure 4a. The lowest κ_T_ was obtained in TiNiSn-1 at. % TiN composition across the whole temperature range. The κ_T_ of TiNiSn-1 at. % TiN is about 5.4 W/mK at 323 K, and decreases as the temperature increases, reaching 4.2 W/mK at 600 K. These values are lower than those reported across the whole temperature range [35,36,37,38,39]. The κ_T_ is comprised of summation of κ_e_ and κ_L_ contributions (κ_T_ = κ_e_ + κ_L_). The κ_e_ could be obtained by the Wiedemann-Franz law, κ_e_ = LT/ρ, where L denotes the Lorenz number. We used a Lorenz number of L = ρκ_e_/T = (π^2^/3)·(k_B_/e)^2^ = 1.6 × 10^−8^ WΩK^−2^, calculated by Muta et al. [40]. The calculated κ_L_ of the sample is represented in Figure 4b as a function of temperature, where κ_L_ is dominant in κ_T_ for TiNiSn alloys. The κ_L_ was significantly decreased with the addition of TiC or TiN in TiNiSn matrix, which is probably caused by the enhancement of point defect phonon scattering from interstitial C or N atoms. However, the exact mechanism of the significant reduction of effective κ_L_ for TiN addition is not clear from this study, and would require further study.

**Figure 5:**
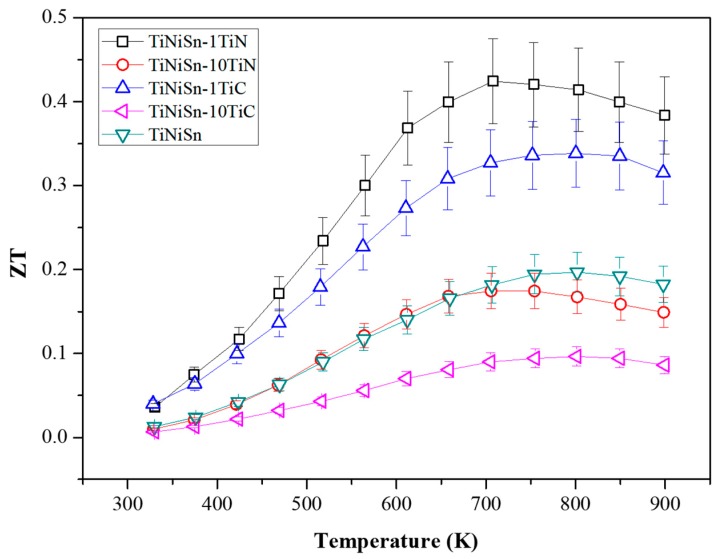
Temperature-dependent figure of merit *ZT* for TiNiSn, TiNiSn-1TiN, TiNiSn-10TiN, TiNiSn-1TiC, and TiNiSn-10TiC.

The TE properties of the TiNiSn alloys are highly dependent on the additive elements. For example, it is thought that the ρ of TiNiSn-10 at. % TiC is completely changed from having semi-conducting properties to having metallic properties, because TiC could be assumed to be partially occupied in the lattice sites of TiNiSn alloys. Also, it is thought that the κ_T_ of TiNiSn-1 at. % TiN is reduced because the precipitation embedded in the TiNiSn matrix, which could be efficient scattering effects of phonons, is negatively affected in terms of electrical resistivity. However, the effects of the addition of TiN for TiNiSn on the TE properties are not fully understood. The amount of TiN and TiC additions for TiNiSn alloys should be chosen and characterized so that the change of the TE properties is understood across the whole temperature range. Figure 5 shows the temperature dependence of the figure of merit *ZT* values of the TiNiSn alloys. The *ZT* values are enhanced by incorporation of 0.1 at. % of TiN or TiC in TiNiSn alloy, where the maximum *ZT* values are obtained 0.43 and 0.34 at 723 K for 0.1 at. % TiN and TiC, respectively.

## 4. Conclusions

TiNiSn-TiN and TiNiSn-TiC alloys were prepared by using arc-melting and annealing. The investigated TiNiSn alloys were poly-crystalline in structure. The transport properties, such as the Seebeck coefficient, suggested that the TiNiSn-TiN alloys were degenerate semiconductors. The extrinsic and the intrinsic region were clearly characterized in the analysis of the Seebeck coefficient. In contrast, the transport properties, such as the Seebeck coefficient, showed metallic behavior in the TiNiSn-TiC alloys. The highest *ZT* value was observed in TiNiSn-1 at. % TiN and reached 0.43 at 723 K, which was mainly due to it having the highest Seebeck coefficient and the lowest thermal conductivity.
